# Formaldehyde in Alcoholic Beverages: Large Chemical Survey Using Purpald Screening Followed by Chromotropic Acid Spectrophotometry with Multivariate Curve Resolution

**DOI:** 10.1155/2011/797604

**Published:** 2011-06-28

**Authors:** Julien A. Jendral, Yulia B. Monakhova, Dirk W. Lachenmeier

**Affiliations:** ^1^Chemisches und Veterinäruntersuchungsamt (CVUA) Karlsruhe, Weissenburger Straße 3, 76187 Karlsruhe, Germany; ^2^Department of Chemistry, Saratov State University, Astrakhanskaya Street 83, Saratov 410012, Russia

## Abstract

A strategy for analyzing formaldehyde in beer, wine, spirits, and unrecorded alcohol was developed, and 508 samples from worldwide origin were analyzed. In the first step, samples are qualitatively screened using a simple colorimetric test with the purpald reagent, which is extremely sensitive for formaldehyde (detection limit 0.1 mg/L). 210 samples (41%) gave a positive purpald reaction. In the second step, formaldehyde in positive samples is confirmed by quantitative spectrophotometry of the chromotropic acid-formaldehyde derivative combined with Multivariate Curve Resolution-Alternating Least Squares (MCR-ALS). Calculation of UV-VIS and ^13^C NMR spectra confirmed the monocationic dibenzoxanthylium structure as the product of the reaction and disproved the widely cited *para,para*-quinoidal structure. Method validation for the spectrophotometric procedure showed a detection limit of 0.09 mg/L and a precision of 4.2–8.2% CV. In total, 132 samples (26%) contained formaldehyde with an average of 0.27 mg/L (range 0–14.4 mg/L). The highest incidence occurred in tequila (83%), Asian spirits (59%), grape marc (54%), and brandy (50%). Our survey showed that only 9 samples (1.8%) had formaldehyde levels above the WHO IPCS tolerable concentration of 2.6 mg/L.

## 1. Introduction

The International Agency for Research on Cancer (IARC) has upgraded the cancer classification of formaldehyde in 2006 to now being clearly “carcinogenic to humans” (group 1) [1]. More recently, formaldehyde has been implicated by the IARC as a causative agent of leukemia as well as nasopharyngeal cancer in humans [2]. The US EPA provides a reference dose for chronic oral exposure (RfD) of 0.2 mg/kg bodyweight/day [3]. The WHO IPCS [4] has established a tolerable concentration (TC) of 2.6 mg/L in ingested products based on animal experiments [5]. Systematic data are currently lacking regarding the formaldehyde content of alcoholic beverages or indeed of most food in general. Feron et al. [6] estimated that the formaldehyde intake by food may range between 1.5 and 14 mg/person/day, which could, therefore, exceed the RfD in a worst case scenario.

**Figure 1:**
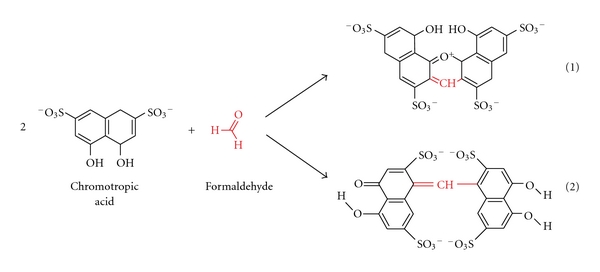
Hypothetical reaction products of the chromotropic acid-formaldehyde reaction [7]. The monocationic dibenzoxanthylium structure (1) is the more likely product (see text).

**Figure 2:**
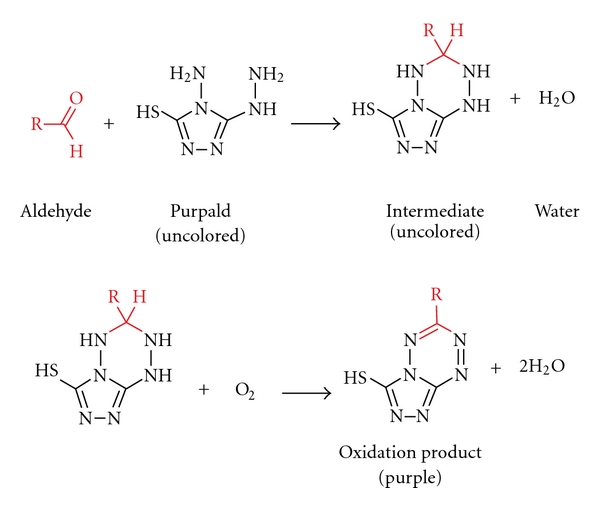
Reaction mechanism for the determination of aldehydes using purpald according to Hopps [8].

As we had previously conducted in-depth research into acetaldehyde content of alcoholic beverages [9, 10], we were recently asked if we had similar data on formaldehyde and if this compound may pose a risk to consumers in addition to the risk of ethanol [11]. We had no such data, because formaldehyde cannot be analyzed along with the other volatiles (e.g., acetaldehyde and methanol) during a typical gas chromatographic reference method [12]. For this reason, a separate assay has to be developed. It quickly became clear that instrumental techniques such as GC [13–20], HPLC [21–30], or flow-injection fluorimetric analysis [31–33], which require extensive sample preparation and derivatization steps, in addition to having costly and complicated instrumental requirements, would not be feasible for the economic and time-efficient survey of a large number of samples. In contrast, the commonly applied AOAC reference method 931.08 [34], based on the chromotropic acid reaction first described by Eegriwe [35], appeared to be applicable (Figure 1). However, it is a comparatively time-consuming method, which meant that the sample throughput would not have been large (our aim was to analyze at least 100 samples to provide a valid exposure assessment for consumers). Therefore, we had the idea to preselect samples for the AOAC procedure by first using a rapid colorimetric screening test for formaldehyde. A number of these tests are available for screening of aldehydes (e.g., Fehling's, Tollen's and Schiff's reagents, see summary by Brandl [36]), but these classical tests suffer from their low specificity. However, as late as 1970, a new test using purpald reagent (4-amino-3-hydrazino-5-mercapto-1,2,4-triazole, CAS# 1750-12-5) was developed, which is remarkably sensitive and specific for aldehydes [8, 37, 38]. The purpald reaction is based on a condensation of formaldehyde with the reagent to form an aminal, which then reacts under aeration (facilitated by vigorously shaking of the solution) to form a purple coloured oxidation product (Figure 2). The reaction is sensitive for aldehydes, as ketones are oxidized to an uncoloured product [8, 37].

Conveniently, the reagents needed for the purpald as well as the chromotropic acid determination are available in ready-to-use test kits (intended for water or disinfectant analysis), and these appear to be usable for testing of alcoholic beverages as well. The major aim of this paper is to evaluate this two-step strategy to provide a survey of formaldehyde in alcoholic beverages. Furthermore, we provide some insight into the structure of the formaldehyde-chromotropic acid chromogen and apply multivariate curve deconvolution techniques to improve the spectrophotometric assay.

## 2. Experimental Section

### 2.1. Instrumentation

All chemicals are commercially available. The formaldehyde test kits (Aquamerck No. 1.08028, based on the purpald reaction, and Spectroquant No. 1.14678, based on the chromotropic acid reaction), as well as the sulphuric acid (95–97%) and absolute ethanol, were purchased from Merck (Darmstadt, Germany). Ortho-phosphoric acid (85%) was obtained from Sigma-Aldrich (Taufkirchen Germany). A formaldehyde standard solution (200 mg/L in 40% vol ethanol) was prepared from a 37% formaldehyde stock solution (Merck, Darmstadt, Germany) and confirmed by iodometric titration (as specified in the application note supplied with the Spectroquant test kit).

A Vortex Genie 2 mixer (Scientific Industries Inc., Bohemia, NY) was used for homogenization of solutions. Sample temperature was controlled in a DC10-W26 heating circulator bath (Haake, Karlsruhe, Germany). A Vapodest 30 automated distillation device (C. Gerhardt, Fabrik und Lager chemischer Apparate, Bonn, Germany) was used for distillation, with condenser cooling at 1°C provided by a recirculating chiller B-740 (Büchi, Flawil, Switzerland). Spectrophotometric measurements were performed on a Perkin Elmer Lambda 12 dual beam spectrometer equipped with an automatic cell changer. The spectrometer was operated with the UV WinLab software (version 2.80.03). The spectra were acquired in a range between 350 and 800 nm at a scanning speed of 120 nm/min with a data interval of 1.0 nm. All measurements were made against ethanol (40% vol) as a blank.

### 2.2. Rapid Screening Using Purpald Reagent

A total of 508 samples of alcoholic beverages (beer, wine, spirits, and unrecorded alcohol; see [39, 40] for details on unrecorded alcohol) were analyzed using the purpald method according to the specifications of the test kit. Briefly, 5 mL of the sample were pipetted into a test tube, and five drops of the alkalinization reagent were added to raise the pH to a level above 13. Afterwards, the reaction was started by adding a microspoonful of the purpald reagent. The solution was then carefully mixed on the vortex mixer. After a reaction time of five minutes, a purple colour indicated a positive result. Uncoloured solutions indicated the absence of any aldehyde (detection limit at 0.1 mg/L according to the manufacturer).

### 2.3. Quantitative Photometric Determination Using the Chromotropic Acid Method

**Figure 3:**
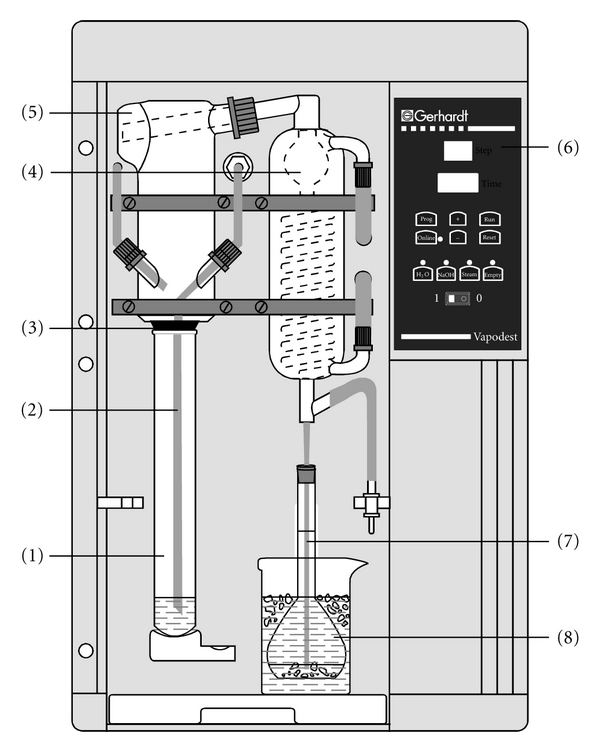
Automated steam distillation device for the determination of formaldehyde in alcoholic beverages: (1) Kjeldatherm digestion tube with acidified sample, (2) steam inlet tubing, (3) Viton-cone, (4) distillation condenser cooled at 1°C, (5) distribution head, (6) keyboard and display, (7) distillate outlet tubing, and (8) cooled graduated flask as receiver containing ice and sulfuric acid (25%), in which the outlet tubing must be submerged to avoid losses.

Only samples that showed a positive purpald reaction needed further analysis. Formaldehyde in these was quantified using the chromotropic acid method. Uncoloured samples (e.g., vodka, blanco tequila, fruit spirits) were used without further preparation. Coloured samples (e.g., beer, wine, whiskey) were distilled according to the AOAC method [34]. The distillation was accomplished using an automated steam distillation device (see [41–43] for further details on steam distillation in the sample preparation of alcoholic beverages). To ensure consistent experimental conditions, the system was preheated before every startup, running a blank sample at full steam power (according to the manufacturer's instructions). Next, 100 mL of sample were brought to 20°C and pipetted into a 250 mL Kjeldatherm digestion tube. To prevent evaporation while multiple tests were prepared, the tubes were temporarily sealed using laboratory film. To prepare the receiver, a spatula of crushed ice was placed in a 50 mL graduated flask before adding 3 mL of sulphuric acid (25%). The receiver was then placed underneath the outlet tubing of the distillation device in a beaker filled with ice water to assure cooling during the distillation process (Figure 3). Just before clamping the digestion tube into the distillation device, 1 mL of phosphoric acid (85%) was added. The automatic distillation was started and run until the calibration mark was nearly reached. After termination, the flask was tightly sealed, the distillate was brought to 20°C in a water bath and filled up to the calibration mark with 40% ethanol.

The chromotropic acid method was performed according to the manufacturer's test kit instructions. A 5 mL volume of the sulphuric reagent was pipetted into a test tube, and a microspoonful of the chromotropic acid reagent was dissolved by holding the tube in an ultrasonic bath for approximately 120 seconds. A 3 mL volume of the sample solution or the distillate was then slowly pipetted into the tube. To start the reaction, the hot tube was closed with a screw cap, and the solution was carefully homogenized using a vortex mixer. After a reaction time of 10 minutes, the contents of the tube were briefly mixed again. A 2.5 mL volume of the reaction liquid was transferred into a cuvette for measurement of the absorbance at a wavelength of 565 nm as well as to record a full spectrum. The calibration was conducted using freshly prepared standards (0.5, 1.0, 2.0, 4.0, 8.0 mg/L in vodka 40% vol) treated similarly to the samples.

### 2.4. Multivariate Analysis to Improve Quantitative Photometry

In preliminary experiments with pure standards, no interference was noted for the chromotropic acid method even if acetaldehyde was present in large excess. However while analysing real samples, we noted interferences of other compounds that led to yellowing/browning of the solution. For many samples, the signals created by these matrix compounds were sometimes relatively large and more or less overlapped the formaldehyde peak. For this reason, calibration on a single wavelength (565 nm) is not recommended for formaldehyde quantification in alcoholic beverages. Instead, we evaluated different multivariate techniques such as multivariate curve resolution (MCR) or independent component analysis (ICA). These methods are able to extract the target spectrum from a complex matrix and therefore could improve the analytical procedure to determine formaldehyde in alcoholic beverages.

Much research in recent years has been done to solve the mixture analysis problem and to extract real spectra and concentration profiles from overlapping spectral data without any *a priori* assumptions about the composition of the system [44–48]. 

Nowadays, the MCR-ALS (Multivariate Curve Resolution-Alternating Least Squares) method developed by Tauler [46, 49, 50] is the most well-known and frequently used self-modelling algorithm for spectral deconvolution [44, 51]. Results of this method can be considerably improved by applying contraints based on the previous knowledge about the system studied (e.g., nonnegativity, closure, unimodality) [46]. Previous examples in food analysis include the determination of vitamins, food colours, alcohols, and dairy products [44, 52–54].

Another set of approaches for multivariate analysis is known as “independent component analysis” (ICA) [55, 56]. In some interpretations, ICA can be considered as an extension of principal component analysis (PCA), the basis of many chemometric methods. ICA methods differ in numerical measures of statistical independence and approximations. The MILCA algorithm (Mutual Information Least Dependent Component Analysis) is employed in the present study. This algorithm possesses some unique properties that make it advantageous in comparison with other ICA techniques [57]. MILCA is based on the search for the least dependent (in contrast to independent) mixture components gauged by precise numerical estimates of mutual information [58] as a measure of signal dependence. It was found that MILCA outperforms other specialized chemometric algorithms for spectra decomposition problems and can be used for, for example, the analysis of vitamins [47, 52] and human brain samples [57].

The third algorithm we apply is SIMPLISMA (Simple-to-use-Interactive-Self-Modelling-Mixture-Analysis) [59], which belongs to the pure variable selection methods. It finds the most representative row or column profiles for the different compounds in the data set. When the selectivity conditions are favourable, the row or column can be directly associated with the pure concentration or response profile and the successful resolution of the mixture can be achieved.

For MCR calculation we used the software Unscrambler X version 10.0.1 (Camo Software AS, Oslo, Norway). We applied nonnegativity constraints during the ALS optimization. For the ICA calculations, we used Matlab v. 7.0 (The Math Works, Natick, Mass, USA). We applied the SIMPLISMA [59] and MILCA [58] algorithms. The data set for analysis comprised of the 321 alcoholic beverages tested positive in the purpald assay. To assess the similarities between the resolved and the experimental spectra, we used Pearson's correlation coefficient (*R*).

### 2.5. Quantum-Chemical Calculations for Structure Elucidation

HyperChem Professional (Hypercube, Gainesville, Fla, USA) software package (v.8.0) was used for quantum chemical calculations. The main goal of all quantum-chemical methods is the solving of the Schrödinger equation. In this case based on the Hartree-Fok-Rutan equation by the self-consistent field (SCF) method, we applied the semiempirical PM3 (Parametrised Model 3) method for calculation with full geometry optimization. In most cases it is the most accurate semiempirical method. The main approaches of the PM3 method include adiabatic, one-electron, MO LCAO (molecular orbital as a linear combination of atomic orbitals) and INDO (Intermediate Neglect of Differential Overlap) approximations. For UV-VIS spectra calculation, we used 5 occupied and unoccupied orbitals using the configuration interaction (CI) method. For details regarding the calculations, see [60].

^13^C NMR spectra calculations were carried out using ChemBioDraw 12.0 software (CambridgeSoft, Cambridge, UK). Chemical shifts are estimated for all hydrogens or carbon atoms for which additivity rules are available. Following a hierarchical list, the algorithm first identifies key substructures of a molecule. A substructure provides the base value for the estimated shift. The ^13^C NMR Shift tool is based on 4000 parameters. It also implements models for ethylenes (cis/trans) and cyclohexanes (equatorial/axial). In case of ^13^C NMR, it estimates over 95% of the shifts with a mean deviation of 0.29 ppm and standard deviation of 2.8 ppm. For details, see [61, 62].

## 3. Results and Discussion

### 3.1. Purpald Screening

**Figure 4:**
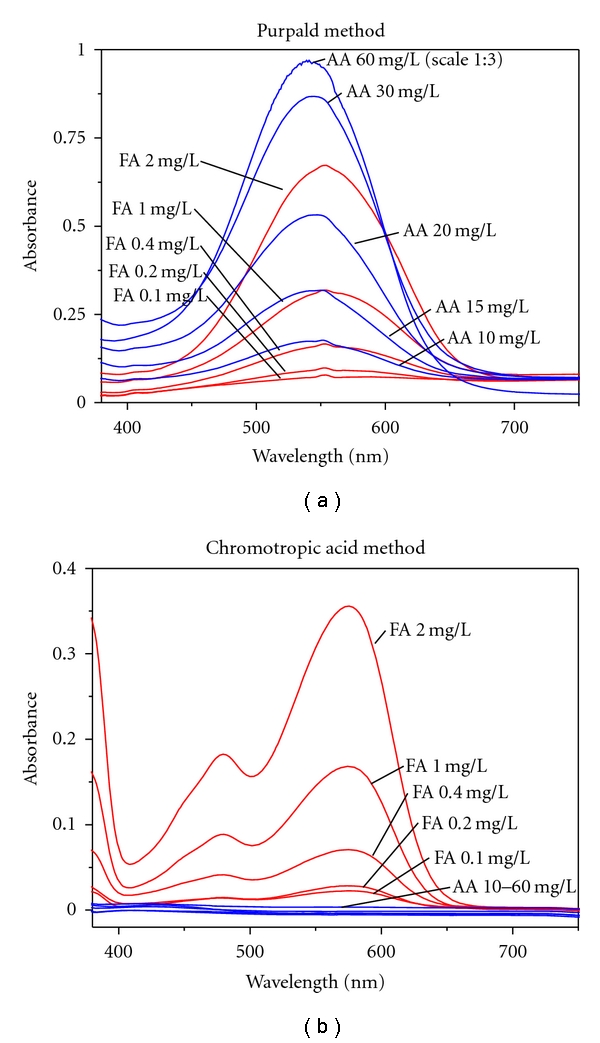
VIS-Spectra of formaldehyde (FA) and acetaldehyde (AA) reaction products with purpald (a) and chromotropic acid (b).

**Figure 5:**
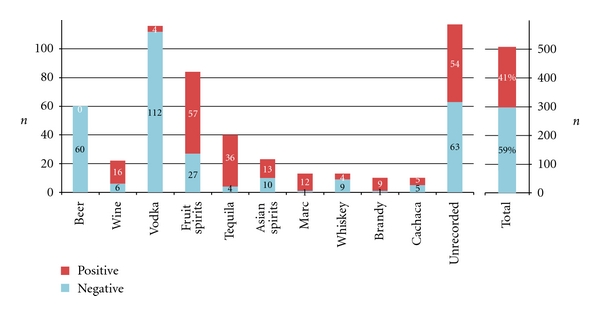
Results of the screening using purpald reagent.

In the first stage of the project, we evaluated the purpald test kit for use with alcoholic beverages (the test kit was originally intended for disinfectant and rinsing solutions (e.g., laundries) and aqueous solutions). We have spiked vodka samples (40% vol) with different concentrations of formaldehyde and acetaldehyde and not only visually examined the colour reaction but also made a spectrophotometric measurement of the full visible light spectrum. Our results show that the purpald assay is usable with alcoholic solutions, and we were able to confirm the manufacturer's detection limit of 0.1 mg/L. While it was recognized that the absorption maxima of different aldehydes are too close to allow their differentiation [38], we are the first to quantitatively record differences in sensitivity between formaldehyde and acetaldehyde (the assay is approximately 20 times more sensitive for formaldehyde than for acetaldehyde (Figure 4(a))). As many alcoholic beverages (i.e., especially beer, vodka, or rum) contain less than 20 mg/L of acetaldehyde [9], the specificity of this assay is suitable to select formaldehyde-positive samples without an unacceptably high number of false-positives. Although we were able to exclude more than 50% of the original samples as being free of formaldehyde or any other aldehyde detectable with purpald (Figure 5), the assay does not allow us to conclude that a product in fact does contain formaldehyde, so that a more specific confirmation is then needed. Nevertheless, we think that the strategy to screen the samples with purpald is worthwhile, as the assay is very quick, and it allowed us to screen over 500 samples in 3 weeks, which is more than what we had originally intended.

### 3.2. Chromotropic Acid Confirmation Analysis: Remarks on Operating Procedure and Detection Limits

To confirm the presence of formaldehyde in the samples, we chose the chromotropic acid procedure, which is suggested by AOAC for general use in food analysis [34]. In contrast to the operating procedure of Li et al. [63], we used an automated steam distillation device, as suggested by Steiner and Länzlinger [64] or Liu [65]. In deviation from most previous protocols, the commercial test kit [66] does not require heating of the reagent solution in a water bath (the heat derived from the dilution of sulphuric acid is sufficient to form the violet dye, as also suggested by Steiner et al. [67] for optimization of the assay). Our detection limit (0.09 mg/L in vodka) is similar to that reported in the specifications of the test kit [66] (0.10 mg/L for 10 mm cells), and to that reported by Donhauser et al. [68] (0.1 mg/L), while Li et al. [63] (0.033 mg/L) and Kleinert and Srepel [69] (0.02 mg/L) reported lower, and Krüger and Holländer [70] (0.25 mg/L) reported slightly higher limits. Of course, techniques using chromatographic separation offer limits in the lower *μ*g/L range, but we feel that our limit is sufficient because it is over a factor of 20 lower than the WHO IPCS tolerable concentration of 2.6 mg/L [4]. It is also lower than the EU limit for total aldehydes in vodka (0.5 g/hL of pure alcohol, which is 2 mg/L for a 40% vol spirit).

### 3.3. Chromotropic Acid-Formaldehyde Reaction: Structure Elucidation

**Figure 6:**
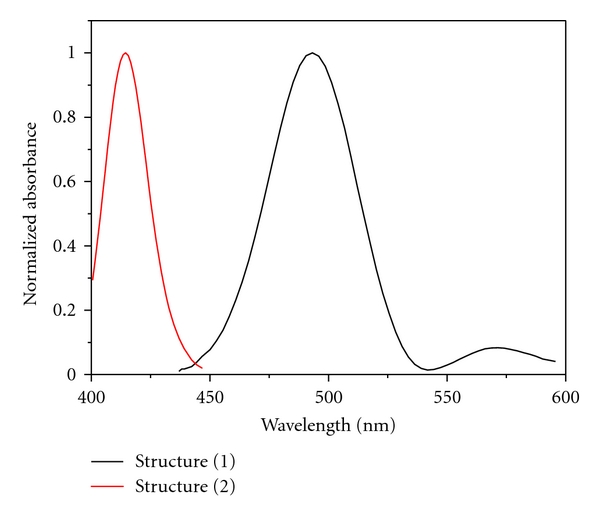
Calculated UV-VIS spectra of the possible reaction products of the chromotropic acid-formaldehyde reaction. Structure (1): monocationic dibenzoxanthylium structure; structure (2): *para*,*para*-quinoidal structure (for structural formulae see Figure 1).

It is rather surprising that the nature of the chromogen of such a widely used analytical procedure such as the reaction of chromotropic acid with formaldehyde has never been unambiguously proven [7]. According to a hypothesis of Georghiou and Ho [7], the actual product would be the monocationic dibenzoxanthylium structure, while the most often quoted structure (e.g., [35, 71]) of the chromogen appears to be the very unlikely *para*,*para*-quinoidal structure (Figure 1). However, as their attempt to crystallize the adduct was unsuccessful, the final structure remains unverified [7]. To decide, which structure is more likely, we did semiempirical quantum chemical calculations of the UV-VIS spectra of the two possible adducts of the reaction (Figure 4(b)). It has been found that the maxima of the spectral bands for the monocationic dibenzoxanthylium structure are 493 and 563 nm, which is very similar to the experimental values (first peak around 470–490 and second peak around 560–580) (Figure 6). The correlation coefficient between the calculated spectrum and the experimental spectral data is relatively high (*R* = 0.81). On the other hand, there is no singlet peak in the region of 560–580 nm for the *para*,*para*-quinoidal structure, and the correlation is considerably lower (*R* = 0.44). Additionally, the spatial situation appears to add to the unlikelihood of the formation of the *para*,*para*-quinoidal structure. Calculation of ^13^C NMR spectra revealed that the monocationic dibenzoxanthylium structure would have two peaks at 119 and 33 ppm that are comparable with experimental data from the literature (119 and 27 ppm [7]). The *para*,*para*-quinoidal structure would have a peak at 115 ppm but no signal around 30 ppm. Our UV-VIS and ^13^C NMR calculations provide evidence that it is most likely that the monocationic dibenzoxanthylium is formed. Our data, therefore, confirm the hypothesis of Georghiou [7].

### 3.4. Chromotropic Acid-Formaldehyde Reaction: Interferences

Regarding the chromotropic acid reaction, the literature is not consistent regarding its specificity towards formaldehyde. The original investigation of Eegriwe [35] found no reaction with acetaldehyde or several other aldehydes and further substances. For determination in alcoholic beverages, it is especially advantageous to have a method in which acetaldehyde, methanol, formic acid, acetic acid, and sugars do not interfere, even if present in the proportion of 10 : 1 or more [72]. The specificity of the chromotropic assay towards formaldehyde has been experimentally confirmed by several authors [63, 69, 73, 74]. Only Ahonen et al. [75] detected an interference of acetaldehyde, leading to a lower finding of formaldehyde (88% at 1 mg/L of formaldehyde if acetaldehyde was contained in the solution in excess). In our experiments, no interference was noted even if acetaldehyde was present in large excess (Figure 4(b)). However, we noted interferences of other compounds that led to yellowing of the solution. The signals created by these compounds were sometimes relatively large and more or less overlapped the formaldehyde peak. For this reason, we do not recommend quantification using calibration on a single wavelength (565 nm), but instead suggest that a multivariate technique has to be used.

### 3.5. Chromotropic Acid-Formaldehyde Reaction: Multivariate Curve Deconvolution and Quantitative Results

**Figure 7:**
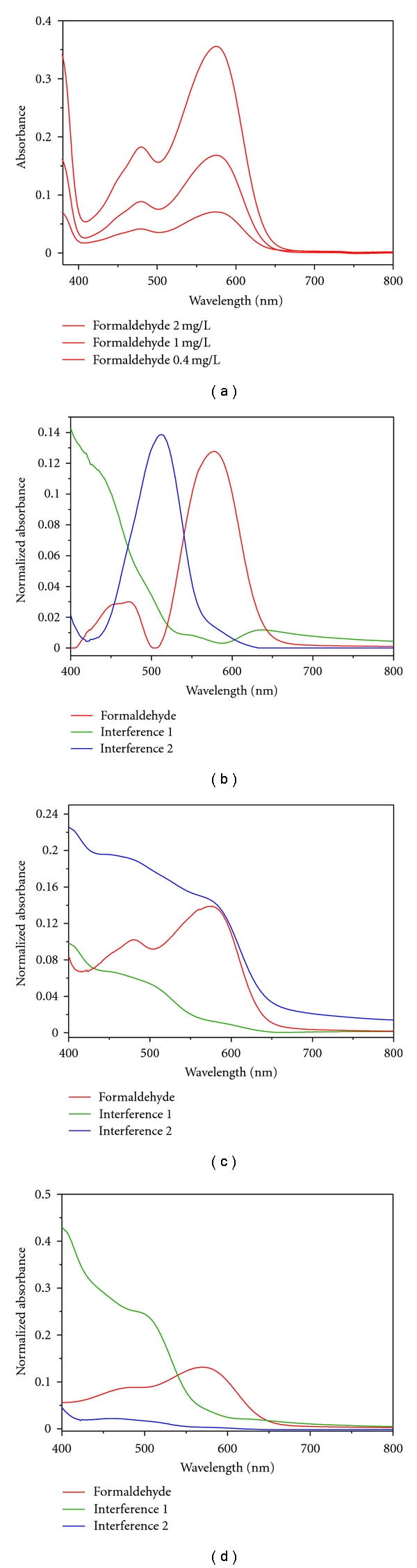
Experimental pure formaldehyde spectra (a) and resolved MCR-ALS (b), MILCA (c), and SIMPLISMA (d) spectra of formaldehyde and two interferences.

The multivariate models that best describe the investigated system consist of three pure compounds for all chemometric methods used. Compared to a set of pure compound spectra (Figure 7(a)), the resolved MCR-ALS spectra are shown in Figure 7(b). It can be seen that one spectrum corresponds to the adduct of formaldehyde-chromotropic acid and the other two belong to interferences. Clearly, the interfering substances strongly absorb at 565 nm and prevent to get accurate results in the single wavelength method. Very similar results were obtained with the two ICA algorithms (Figures 7(c) and 7(d)).

To compare the performance of the different algorithms, we calculated the correlation coefficients (*R*) between the experimental spectrum of the adduct of formaldehyde-chromotropic acid reaction and the resolved signals. The SIMPLISMA algorithm gives the best estimation of the spectral signal (*R* = 0.97). However, ALS (*R* = 0.90) and MILCA (*R* = 0.95) are equally suitable for extracting the pure formaldehyde spectrum. For our final evaluation of the samples (Table 1), we decided to use MCR-ALS as this is implemented in our standard statistical software package.

The precision and accuracy of the method were sufficient for the purpose. The coefficient of variation (CV) for spiked vodka (*n* = 6) was 8.2% at 1 mg/L, 4.2% at 4 mg/L, and 7.9% at 8 mg/L; the recovery for spiked vodka was 101% at 1 mg/L, 99% at 4 mg/L, and 99% at 8 mg/L. For an authentic Asian spirit containing 6.4 mg/L of formaldehyde, the CV was 4.6% (*n* = 6). For the purpose of this survey, we have not conducted further validation studies in other matrices, but would recommend to study the measurement uncertainty in more detail if results should be used in expert opinions (e.g., in legal cases against manufacturers if limits are exceeded).

In total, formaldehyde was confirmed in 132 samples (Table 1). The false positive rate of the purpald assay was 37%. The highest incidence was found in tequila (83%), Asian spirits (59%), grape marc (54%), and brandy (50%). Interestingly, formaldehyde levels were not correlated with ethanol (*P* = .51), acetaldehyde (*P* = .24), or methanol (*P* = .94) levels that had been determined previously using reference procedures [12].

We think that the chromotropic acid reaction along with multivariate curve deconvolution is applicable for the purpose to provide a fast and cheap analysis, for example, in the context of high-throughput screening for occurrence and exposure assessment. We think that only in the case of very high contents of formaldehyde, which would exceed international levels (e.g., the WHO IPCS tolerable concentration (TC) of 2.6 mg/L [4] in ingested products), additional chromatographic confirmatory analysis is needed prior to taking measures against producers.

## 4. Conclusions

In this paper we show how it is possible to quickly survey a large number of samples using a two-step procedure: purpald screening followed by quantitative spectrophotometry using chromotropic acid. We can confirm the suitability of the chromotropic acid reaction for the determination of formaldehyde in alcoholic beverages, giving results similar to those of Li et al. [63]. It is no wonder that the chromotropic acid method is still widely used, as it is simple and inexpensive [76]. As we have shown, the procedure can be improved by using multivariate curve deconvolution, which expands its use to matrices that would be normally excluded due to spectral interferences. In our case, we would go so far to say that the determination of formaldehyde in alcoholic beverages using chromotropic acid is not possible without a chemometric method. The advantage of the overall procedure is that it is simple, reliable and cheaper than chromatographic methods. 

It should be noted that this approach for the determination of a target compound in a complex matrix with interferences is transferable to other similar spectrophotometric problems. Chemometric approaches can be used for any reaction where the signal of the target compound overlaps with interferences. We expect that, as these methods become more and more integrated into standard statistical software packages, their use will considerably increase in the future.

Our survey of about 500 products showed that only 1.8% of the samples had formaldehyde levels above the WHO IPCS tolerable concentration. A 60 kg person would need to consume 0.8 L of alcohol at 14.37 mg/L daily to exceed the US EPA RfD of 0.2 mg/kg bodyweight/day, which is extremely unlikely even in this worst-case scenario. While a more detailed population based risk assessment is needed, which also should include other foods, we preliminarily conclude that formaldehyde is unlikely to pose an additional risk for the alcohol drinking population.

## Figures and Tables

**Table 1:** Results of the quantitative determination using chromotropic acid.

Sample category	Total number tested	Positive samples	Average (mg/L)	Standard deviation (mg/L)	P90 (mg/L)	P95 (mg/L)	P99 (mg/L)	Maximum (mg/L)	Samples above limit (2.6 mg/L)
Beer	60	0%	—	—	—	—	—	—	0
Wine	22	41%	0.13	0.29	0.37	0.54	1.03	1.15	0
Vodka	115	0%	—	—	—	—	—	—	0
Fruit spirits	85	44%	0.20	0.61	0.39	0.68	1.77	5.39	1
Tequila	40	83%	0.70	1.22	1.77	2.87	5.26	6.06	2
Asian spirits	23	59%	2.26	4.60	9.75	13.44	14.21	14.37	4
Marc	13	54%	0.49	0.86	1.66	2.20	2.64	2.75	1
Whiskey	13	31%	0.20	0.46	0.55	1.03	1.50	1.62	0
Brandy	10	50%	0.09	0.61	1.15	1.41	1.62	1.67	0
Cachaça	10	20%	0.10	0.26	0.21	0.51	0.76	0.99	0
Unrecorded	117	29%	0.22	0.71	0.72	1.08	1.56	6.71	1

Total sample	508	26%	0.27	1.21	0.70	1.11	6.14	14.37	9
